# Identification of Susceptibility Genes of Adult Asthma in French Canadian Women

**DOI:** 10.1155/2016/3564341

**Published:** 2016-05-04

**Authors:** Jean-Christophe Bérubé, Nathalie Gaudreault, Emilie Lavoie-Charland, Laura Sbarra, Cyndi Henry, Anne-Marie Madore, Peter D. Paré, Maarten van den Berge, David Nickle, Michel Laviolette, Catherine Laprise, Louis-Philippe Boulet, Yohan Bossé

**Affiliations:** ^1^Institut Universitaire de Cardiologie et de Pneumologie de Québec, Quebec City, QC, Canada G1V 4G5; ^2^Département des Sciences Fondamentales, Université du Québec à Chicoutimi, Chicoutimi, QC, Canada G7H 2B1; ^3^The University of British Columbia Center for Heart Lung Innovation, St. Paul's Hospital, Vancouver, BC, Canada; ^4^University of British Columbia Department of Medicine, Division of Respiratory Medicine, Vancouver, BC, Canada V5Z 1M9; ^5^University Medical Center Groningen, GRIAC Research Institute, University of Groningen, Groningen, Netherlands; ^6^Merck Research Laboratories (MRL), Seattle, WA, USA; ^7^Department of Molecular Medicine, Laval University, Quebec City, QC, Canada G1V 0A6

## Abstract

Susceptibility genes of asthma may be more successfully identified by studying subgroups of phenotypically similar asthma patients. This study aims to identify single nucleotide polymorphisms (SNPs) associated with asthma in French Canadian adult women. A pooling-based genome-wide association study was performed in 240 allergic asthmatic and 120 allergic nonasthmatic women. The top associated SNPs were selected for individual genotyping in an extended cohort of 349 asthmatic and 261 nonasthmatic women. The functional impact of asthma-associated SNPs was investigated in a lung expression quantitative trait loci (eQTL) mapping study (*n* = 1035). Twenty-one of the 38 SNPs tested by individual genotyping showed *P* values lower than 0.05 for association with asthma.* Cis*-eQTL analyses supported the functional contribution of rs17801353 associated with* C3AR1* (*P* = 7.90*E* − 10). The asthma risk allele for rs17801353 is associated with higher mRNA expression levels of* C3AR1* in lung tissue.* In silico* functional characterization of the asthma-associated SNPs also supported the contribution of* C3AR1* and additional genes including* SYNE1*,* LINGO2*, and* IFNG-AS1*. This pooling-based GWAS in French Canadian adult women followed by lung eQTL mapping suggested* C3AR1* as a functional locus associated with asthma. Additional susceptibility genes were suggested in this homogenous subgroup of asthma patients.

## 1. Introduction

Substantial efforts have been deployed to discover the genetic variants associated with asthma [1, 2]. Asthma constitutes a considerable burden for individuals and health services with more than 300 million persons affected worldwide [3]. Various approaches have been used to identify asthma risk loci and genome-wide association studies (GWASs) have discovered the most robust genetic associations [4, 5]. This genomic approach still requires substantial resource in terms of sample size and genotyping. To reduce the genotyping burden, an alternative methodology has been developed, which consists of a GWAS on pooled DNA samples (pooled GWAS) followed by further validation of the top associations by individual genotyping. This approach has been shown to be effective for several complex traits [6]. It has also been applied to asthma and has confirmed known asthma loci and led to the identification of new ones [7, 8].

GWAS SNPs discovered so far account for a relatively low percentage of the total asthma heritability. Studying more homogeneous subgroups of asthma patients is likely to reveal part of this “missing heritability.” The rationale is that individuals within subgroups are more likely to share the same underlying molecular basis. It is known that asthma prevalence differs between men and women throughout life [9]. This sex difference is not completely understood. Studying the genetics of asthma by gender and age groups is thus important [10].

In this study, we used a pooled GWAS to detect SNPs associated with asthma in French Canadian women enrolled in the Quebec City Case-Control Asthma Cohort (QCCCAC). Confirmation by individual genotyping was followed by analyses of expression quantitative trait loci (eQTL) in human lung tissues,* in silico* analyses for functional prediction and validation in a second collection of French Canadian women.

## 2. Methods

**Figure 1:**
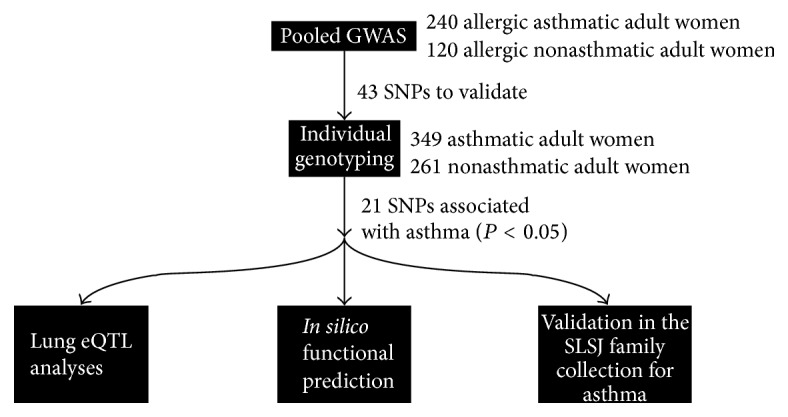
Overview of the experimental design.

The experimental design is summarized in Figure 1.

### 2.1. Participants

Cases and controls included in this study are part of the Quebec City Case-Control Asthma Cohort [11]. Briefly, the QCCCAC consists of unrelated adults of self-reported European ancestry recruited at the research center of the* Institut Universitaire de Cardiologie et de Pneumologie de Québec* (IUCPQ). All research participants were ≥18 years old at enrollment. Individuals with chronic obstructive pulmonary disease (COPD), body mass index >40 kg/m^2^, and/or any systemic inflammatory disease were excluded. Individuals with self-reported genetic relatedness were also excluded. Participants provided written informed consent and the study was approved by the ethics committee of the IUCPQ. For the current study, only women were investigated and the asthma diagnosis was confirmed by a respirologist based on clinical symptoms, lung function, and airway responsiveness. Twenty-five inhalant allergens were evaluated by skin-prick tests to measure the allergic status. Participants were considered atopic if at least one allergen caused a wheal diameter of at least 3 mm at 10 min in the presence of a negative saline control and a positive histamine response. Asthma-associated SNPs were tested for replication in women participants of the Saguenay-Lac-Saint-Jean (SLSJ) asthma family collection (*n* = 353). Further details are provided in Supplementary Material available online at http://dx.doi.org/10.1155/2016/3564341.

### 2.2. Pooled GWAS and Analysis

DNA extraction and sample pooling methods are provided in Supplementary Material. Both pools (i.e., cases and controls) were genotyped in 6 replicates using the Illumina HumanOmniExpress BeadChip (Supplementary Figure 1). This SNP array interrogated 730,525 SNPs throughout the genome. The SNP array probes' intensities were formatted and analyzed by the GenePool software [12]. GenePool ranked SNPs by increased likelihood of being genetically associated with asthma. To do so, the Manhattan distance method and the silhouette score clustering method were used as implemented in GenePool. Each SNP was assigned a silhouette score that ranged from 0 to 1. A silhouette score of 1 indicates that the allele frequencies for a specific SNP are unequivocally different between cases and controls. Further details are provided in Supplementary Material.

### 2.3. Individual Genotyping and Genetic Association Tests

Following the pooled GWAS, a total of 43 SNPs were selected for validation by individual genotyping. First, the 20 SNPs with the best silhouette score were included. Then, SNPs ranked in the top 2000 of the pooled GWAS and found in or near (50 kb) genes previously associated with asthma, COPD, or related phenotypes in candidate gene studies and GWAS were selected [2, 13]. This resulted in 23 additional SNPs for validation by individual genotyping. Selected SNPs (*n* = 43) were genotyped in an extended cohort of 349 asthmatic women and 261 nonasthmatic women derived from the QCCCAC. This extended cohort contained the women used in the pooled GWAS. Genotyping and quality controls are provided in Supplementary Material. After quality control, 38 out of the 43 SNPs remained. Association tests were done using additive logistic regression models as implemented in PLINK v1.07 [14]. Analyses were first performed in allergic cases (*n* = 299) and allergic controls (*n* = 154) women to mirror the selection of individuals used in the pooling-based GWAS. Analyses were then repeated in all available cases (*n* = 349) and controls (*n* = 261) women with or without allergy in an attempt to increase sample size and evaluate the specificity of genetic signals. *P* value <0.05 was considered suggestive evidence of association. *P* value that passed Bonferroni correction (0.05/38 = 0.0013) was considered statistically significant.

### 2.4. eQTL and* In Silico* Analyses

The functional effects of the asthma-associated SNPs were determined by examining their effect on gene expression in human lung tissue. The lung expression quantitative trait loci (eQTL) mapping study has been previously described [15, 16]. Briefly, 1111 lung specimens were obtained from patients who underwent lung resection at three sites: Laval University (Quebec City, Canada), University of Groningen (Groningen, Netherlands), and University of British Columbia (Vancouver, Canada). Whole-genome gene expression and genotyping data were obtained from these specimens. The genotyping platform used in the eQTL dataset is the Illumina Human1M-Duo BeadChip. The imputation was performed with SHAPEIT v2 and IMPUTE v2 using the 1000-genome project phase 1 data as a reference set. In the present study, SNPs associated with asthma, including SNPs in linkage disequilibrium (LD), were tested for association with gene expression in the Laval dataset (*n* = 407). Replication was then performed in the Groningen (*n* = 341) and UBC (*n* = 287) cohorts.* In silico* analyses were also performed to investigate the functional impact of asthma-associated SNPs. Tools used in this study are Combined-Annotation-Dependent Depletion (CADD) V1.3 [17], SNP Function Prediction (FuncPred) [18], RegulomeDB V1.1 [19], and Haploreg V4 [20]. Further details are provided in Supplementary Materials.

## 3. Results

### 3.1. Subjects

A total of 240 asthma patients and 120 controls were considered in the pooled GWAS. All subjects were atopic women with mean age of 34.0 ± 13.9 years for cases and 35.2 ± 14.3 years for controls, respectively. Individual genotyping was performed in an extended cohort of 349 asthmatic women and 261 controls. Clinical characteristics are summarized in Table 1.

### 3.2. Pooled GWAS

**Figure 2:**
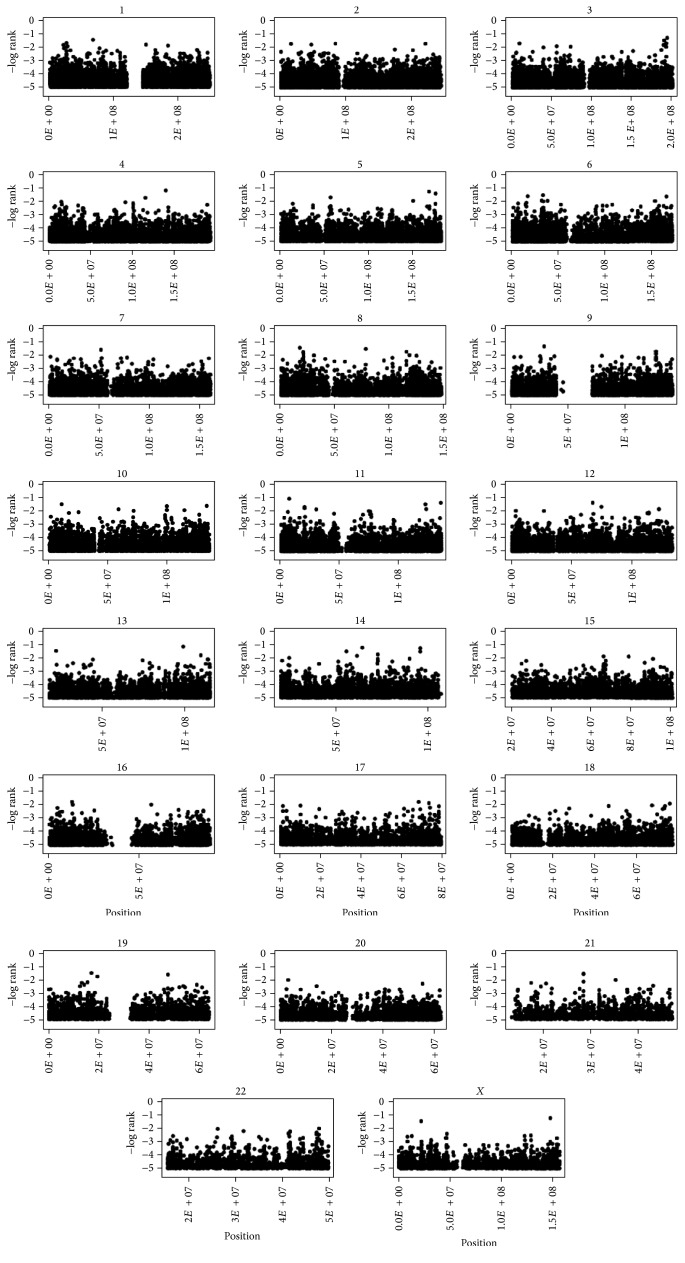
Genome-wide plot of the silhouette scores for the top 100,000 SNPs. The chromosome number is indicated on the top of each subgraph. The *y*-axis represents the silhouette score ranking on a log scale and the *x*-axis indicates the position of the SNPs in physical distance.

Genotyping data from DNA pools were analyzed with the GenePool software. All SNPs were ranked according to the likelihood of being genetically associated with asthma using the silhouette score (Figure 2). The top 20 SNPs selected for individual genotyping are shown in Table 2 and ranked by the silhouette score. One SNP, rs17093106, failed the Illumina design assay step for individual genotyping and was replaced by the 21st top SNP, rs17500510. The SNP rs12418753 was ranked first with a silhouette score of 0.709. The silhouette scores in Table 2 ranged from 0.636 to 0.709. All SNPs were located in noncoding regions except SNP rs12881815 located in an exon of the* SYNE2* gene. None of these SNPs had been previously associated with asthma. Twenty-three additional SNPs located near or within asthma candidate genes were well ranked (i.e., top 2000) and selected for individual genotyping (Table 3).

### 3.3. Individual Genotyping

From the 43 selected SNPs that were typed by individual genotyping, 38 passed quality controls. For the top 20 ranked SNPs in the pooled GWAS, 18 SNPs passed the quality controls. The genotyping assay failed for the only exonic SNP, rs12881815, and for rs12493799. For the 23 SNPs near candidate genes among the top 2000 ranked SNPs, 20 passed the quality controls.

The association with asthma was first tested in atopic women only to mirror the selection of women included in the pooled GWAS. Associations with asthma were suggestive for 29 SNPs out of 38 (76%) and 7 of them were significant after Bonferroni correction (Supplementary Table 1). All 7 SNPs were part of the top ranked SNPs in the pooled GWAS. For the SNPs located near candidate genes and among the top 2000 ranked SNPs, 13 showed a suggestive evidence of association. Overall, only 9 SNPs had *P* values >0.05, ranging from 0.06 to 0.23.

The same 38 SNPs were then tested in an extended cohort containing all available cases and controls women genotyped in the QCCCAC. Twenty-one out of the 38 SNPs (55%) demonstrated suggestive associations with asthma (Table 4). Three SNPs were significant after correction for multiple testing including two from the top ranked SNPs and one from the SNPs near candidate genes. Association tests were also performed in 255 men (213 cases, 142 controls) of the QCCCAC and all suggestive associations were stronger in women compared to men (Supplementary Figure 2). The two strongest associations involved two SNPs newly implicated in asthma, rs17655581 and rs7980829, located near* SLC15A1* and in an intergenic region, respectively. In general, new SNPs identified in the top of the pooled GWAS showed stronger associations with asthma compared to those selected due to their proximity to candidate genes. However, three SNPs near candidate genes were part of the strongest associations. They were rs803010 near* PTGDR* and* PTGER2*, rs10932034 near* ICOS*, and rs17453235 near* DPP10*. Results for the 21 SNPs with *P* < 0.05 for association with asthma and those in LD are detailed in Supplementary Table 2, including information on the genomic context, the LD mapping, the pooled GWAS, the individual genotyping in all case and control women, the replication in SLSJ family collection, and the* in silico* functional prediction.

### 3.4. Lung eQTL Analyses

**Figure 3:**
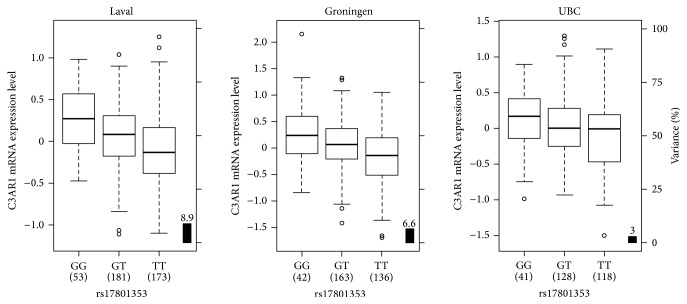
Boxplots of lung mRNA expression levels for* C3AR1* according to genotype groups for SNP rs17801353 in lung tissues from Laval (*n* = 407), Groningen (*n* = 341), and UBC (*n* = 287). The right *y*-axis shows the proportion of the gene expression variance explained by the SNP (black bar). The *x*-axis represents the three genotyping groups for SNP rs17801353 with the number of subjects in parenthesis.

To investigate the potential function of SNPs associated with asthma in the QCCCAC, we analyzed a large-scale lung eQTL dataset [15]. SNPs in LD (*n* = 309) with the 21 asthma-associated SNPs were tested in the eQTL dataset. Fifty-five out of these SNPs were genotyped in the lung eQTL dataset and the genotype information for the remaining SNPs was obtained by imputation. Each of them was tested against the expression levels of all noncontrol probesets interrogated by the gene expression microarray (*n* = 51,627). The most significant lung eQTL-SNP was rs75871129, which passed the Bonferroni correction for multiple testing threshold (3.1*E* − 9) and was associated with a probeset interrogating* C3AR1* (*P* = 4.06*E* − 10). The SNP was in perfect LD with the asthma-associated SNP rs17801353, which had a similar association with mRNA* C3AR1* levels (*P* = 7.90*E* − 10). All the SNPs in this LD block (*R* ^2^ > 0.8) were also associated with mRNA expression of* C3AR1* in the lung.  Figure 3 illustrates the lung eQTL rs17801353-*C3AR1* in Laval as well as replications in Groningen and UBC. rs17801353 is located in an intron of* FOXJ2* 7.6 kb downstream of* C3AR1*. The eQTL was significant and showed the same direction of effect in the two replication cohorts (*P* < 0.05). The asthma risk allele for rs17801353 in the QCCCAC corresponds to the allele associated with higher mRNA expression levels for* C3AR1*, suggesting that upregulation of this gene may increase asthma susceptibility. The most significant lung eQTL (*P* < 10*E* − 5) are shown in Supplementary Table 3.

### 3.5. *In Silico* Functional Characterization

We investigated if any of the LD SNPs were potentially implicated in regulatory mechanisms using bioinformatics tools. First, we employed CADD to score the deleteriousness of the 309 LD SNPs. A scaled *C*-score higher than 10 was obtained for twenty-five of them, indicating that they are predicted to be in the 10% most deleterious genetic variants of the human genome. Of these 25 SNPs, six are part of LD block located in an intronic region of the gene* LINGO2* on chromosome 9p21.1. SNP rs2295190 located on chromosome 6q25.1 showed the greatest scaled *C*-score (24.5), implying that it is among the top 0.5% most deleterious SNPs of the human genome. This SNP is in LD with rs6934016 located near the* ESR1* asthma susceptibility gene. The scaled *C*-score of each LD SNP is shown in Supplementary Table 2.

The FuncPred software recognised 184 out of the 309 LD SNPs and revealed regulatory predictions (Supplementary Table 4). The intronic SNP rs13081182 showed the strongest regulatory potential score (0.406). This SNP is in LD (*R* ^2^ = 0.94) with SNP rs17016738 associated with asthma in this study (Table 4). The nonsynonymous SNP rs2295190 also showed a strong regulatory potential score (0.395) and is implicated in splicing events. Located in* SYNE1*, this SNP was found to be probably damaging for* SYNE1* based on PolyPhen v2 with a score of 0.994/1.00 (sensitivity: 0.69; specificity: 0.97). This SNP is in LD (*R* ^2^ = 0.85) with SNP rs6934016 associated with asthma in this study (Table 4). The asthma risk allele (A) for rs2295190 corresponds to a missense change that occurs at position 8741 of the protein (L8741M). The FuncPred tool also revealed SNPs with high regulatory potential scores in the LD block located in an intronic region of gene* LINGO2* on chromosome 9p21.1, the same LD block mentioned above with several high scaled *C*-score SNPs.

The RegulomeDB V1.1 attributed a valid score to 276 LD SNPs according to the number of regulatory elements they influence (Supplementary Table 5). Two SNPs in the same haplotype block of chromosome 12p13.31, rs10846377 and rs7955798, were ranked highest with important supporting data. The regulatory prediction for rs10846377 was supported by data including eQTL, transcription factor binding, any motif, and DNase peak score. rs7955798 was supported by eQTL and transcription factor binding/DNase peak. These two SNPs were associated with mRNA expression levels of* C3AR1* in circulating monocytes [21]. Both SNPs are in the same haplotype block identified by the lung eQTL analyses. Furthermore, the second most significant association following individual genotyping, rs7980829, was ranked third in the scores attributed by RegulomeDB V1.1. Many motifs are predicted to be altered by this variant and it is thus likely to affect binding of transcription factors in this region.

The Haploreg V4 software recognised 283 out of the 309 LD SNPs and indicated that several LD SNPs were associated with mRNA expression levels (Supplementary Table 6). The rs7980829 and rs11177020 have been previously associated with the mRNA expression levels of* IFNG-AS1* in various tissues including lymphoblastoid cell lines of Europeans. The LD SNPs on chromosome 12p13.31 were also shown to be associated with mRNA expression levels of* C3AR1* in whole blood.

### 3.6. Validation in the SLSJ Asthma Family Collection

To confirm the genetic associations detected in the QCCCAC, the 21 asthma-associated SNPs were analyzed in the SLSJ asthma study. Clinical characteristics of cases and controls are indicated in Table 1. Eleven out of the 21 SNPs were directly genotyped. None of these SNPs was associated with asthma (Supplementary Table 7). The lowest *P* value for association with asthma was with rs17016738 (*P* = 0.080). The remaining 10 SNPs were tested with proxies if available. None of them showed association with asthma (Supplementary Table 8).

## 4. Discussion

This pooled GWAS was performed on a relatively homogeneous subgroup of asthma patients defined by age, atopic status, ethnicity, and gender (i.e., adult, atopic, French Canadian women with doctor diagnosed asthma). Several loci were associated with asthma and confirmed by individual genotyping in an extended sample of French Canadian women. The functional meaning of asthma-associated SNPs was then evaluated in a large lung eQTL study, which supported* C3AR1* as an asthma susceptibility gene.* In silico* analyses also supported* C3AR1* as well as additional genes neighbouring functional asthma-associated SNPs.

Several GWASs have been performed to study the genetics of asthma [4, 5]. GWAS revealed numerous susceptibility loci that explain only a small fraction of the total asthma heritability. GWASs have the potential to reveal additional loci using extended study design [22]. Studying subgroups of phenotypically similar asthma patients instead of the traditional broad case-control format may reveal new susceptibility loci. Using this strategy, Bønnelykke et al. identified* CDHR3* as a new susceptibility gene in childhood asthma with severe exacerbations [23]. Similarly, we used a homogeneous subgroup of asthma patients. Pooled genotyping was used as it is an economic approach to perform a preliminary screen for evidence of association. As recommended, this screen was followed by individual genotyping for confirmation [24]. SNPs identified with the pooled GWAS were in large part (76%) validated by individual genotyping in atopic women. This pooling-based approach has been used previously to study asthma and new risk loci were identified [7, 8]. These two studies were conducted in allergic asthmatic children of white European descent and in a Chinese population, respectively. None of their findings were replicated in this study, which is likely explained by clinical, demographic, and genetic differences.

In this study, one functional SNP located 7.6 kb from* C3AR1* was associated with asthma. rs17801353 was associated with asthma in the extended case-control sample (*P* = 0.028). Lung eQTL analysis demonstrated that rs17801353 is associated with expression levels of* C3AR1*. This SNP was selected for individual genotyping owing to its proximity with that candidate gene on chromosome 12p13.31. This eQTL is reported for the first time in lung tissue and demonstrated the functionality of rs17801353. However,* in silico* analyses identified a study conducted in circulating monocytes where two SNPs in LD with rs17801353 were found to act as eQTL for* C3AR1* [21].* C3AR1* is involved in the pathogenesis of asthma via the complement system [25, 26]. The expression of this receptor is increased during asthmatic lung inflammation [27]. The same direction of effect is observed in the current study as the asthma risk allele increased the mRNA expression levels of* C3AR1* in the three cohorts, which is also consistent with the study on circulating monocytes.

The two strongest associations with asthma after individual genotyping in the QCCCAC were with rs17655581 and rs7980829. The first one is located on chromosome 13q32.3 at 5.1 kb 3′ of* SLC15A1*. This SNP received no clear prediction for putative function from the bioinformatic tools employed and was not implicated in any significant eQTL. The closest gene,* SLC15A1*, is not known to be involved in asthma but is known to cause inflammation in the intestine by the mediation of intracellular uptake of bacterial products [28]. For rs7980829, located on chromosome 12q15, RegulomeDB V1.1 predicted that this SNP may influence protein binding. Furthermore, Haploreg V4 showed that the SNP is associated with the long intergenic noncoding RNA (lincRNA) expression levels of* IFNG-AS1*. This lincRNA has been shown to influence and regulate the expression of* IFNG* [29, 30]. Interestingly,* IFNG* is an inflammatory cytokine implicated in the pathophysiology of asthma [31].

One LD block located in an intronic region of the gene* LINGO2* on chromosome 9p21.1 contained the most potentially deleterious SNPs predicted by* in silico* analyses. This leucine-rich repeat and Ig domain-containing 2 (*LINGO2*) gene is not known to be implicated in asthma. A recent study found that SNPs flanked by* LINGO2* were associated with airway responsiveness in chronic obstructive pulmonary disease [32]. These SNPs are not in LD with any of the SNPs in the LD block identified in this study (all *R* ^2^ < 0.05).

Among the LD SNPs, only rs2295190 was located in an exon and PolyPhen 2 predicted this missense variant as probably damaging for SYNE1. The asthma risk allele for rs6934016 (A) identified by individual genotyping corresponds to the allele in LD with SNP rs2295190 (T) producing a missense change.* SYNE1* has never been associated with asthma, but two intronic SNPs of this gene have been associated with forced vital capacity according to the Phenotype-Genotype Integrator (PheGenI) [33].* SYNE1* is expressed in skeletal and smooth muscle, particularly in the sarcomeres [34]. Thus,* SYNE1* could potentially have an effect on airway smooth muscle and influence muscle function during asthma attacks. Interestingly, one missense SNP in a related gene,* SYNE2*, was significant in the pooled GWAS but failed individual genotyping.* SYNE1* and* SYNE2* are known to be implicated in several diseases such as lung cancer [35]. Their role in the physiopathology of asthma remains to be confirmed.

Associations were revealed with SNPs among the top 2000 ranked SNPs and near candidate genes. The third most important association with asthma was found with rs803010 (*P* = 4.4 × 10^−4^) located in the promoter of the prostaglandin D2 receptor (*PTGDR*). This gene is known to be involved in asthma in Caucasian populations [36, 37]. PTGDR is activated by PGD_2_ and leads to an increase of intracellular cAMP [38]. This augmentation is notably associated with more pronounced Th2 inflammation [39].* PTGDR* may be implicated in bronchial hyperreactivity since knockout mice (Ptgdr^−/−^) are protected against ovalbumin-induced hyperreactivity [40].

Genetic associations observed in the QCCCAC were not replicated in SLSJ asthma family collection. This could be partly due to the relatively small sample size of both datasets. Attempt to replicate was worthy as these two cohorts share similar age, asthma definition, and genetic background (both being French Canadian). The analyses were also restricted to women. However, asthma heterogeneity, not captured by our study design, may also be responsible for the lack of replication.

The main limitation of this study is the small sample size in which we performed the initial pooled GWAS screen and individual genotyping. However, the identification of loci known to be associated with asthma demonstrated the validity of our study and lends credence to the findings of novel loci. The value and innovation of this study were to investigate the genetic factors involved in a phenotypically similar group of asthma patients. Only asthmatic and nonasthmatic women with allergy were selected for the pooled GWAS. This strategy is likely to be more powerful and to require smaller sample size in order to identify the genetic factors underpinning asthma.

In summary, we used pooling-based GWAS to study asthma in a homogeneous population of adult French Canadian women. We then took advantage of a large-scale lung eQTL dataset and bioinformatics tools to examine the functional significance of our discoveries. Our data supported the potential role of* C3AR1* in asthma. This study also suggests a potential role for new loci, namely,* SYNE1*,* LINGO2*, and* IFNG-AS1*, in the pathogenesis of asthma.

## Supplementary Material

Supplementary materials contain additional details about the methodologies. It also contains two figures showing the pool-GWAS design (Suppl. Figure 1) and the asthma genetic association results stratified by sex in the QCCCAC for the 38 genotyped SNPs (Suppl. Figure 2). Eight supplementary tables are also available showing results for 38 SNPs tested by individual genotyping in 299 allergic cases and 154 allergic controls (Suppl. Table 1), completed results for the 21 SNPs associated with asthma and SNPs in LD (Suppl. Table 2), most significant lung eQTL (Suppl. Table 3), in silico analyses results (Suppl. Tables 4 to 6), and results from the SLSJ asthma family collection (Suppl. Tables 7 and 8).

## Figures and Tables

**Table 1:** Clinical characteristics of women.

Characteristics	Pooled GWAS			Individual genotyping			SLSJ asthma family collection		
	Cases (*n* = 240)	Controls (*n* = 120)	*P* values	Cases (*n* = 349)	Controls (*n* = 261)	*P* values	Cases (*n* = 233)	Controls (*n* = 120)	*P* values
Age (years)	34.0 ± 13.9	35.2 ± 14.3	0.456	35.5 ± 14.8	36.0 ± 14.8	0.691	30.6 ± 17.8	40.6 ± 19.4	<0.0001
BMI (kg/m^2^)	25.3 ± 4.8	24.4 ± 4.1	0.066	25.9 ± 5.4	24.5 ± 4.5	0.001	24.5 ± 6.8	24.4 ± 6.0	0.887
FEV_1_ (% predicted)	95.1 ± 16.5	105.6 ± 11.7	<0.0001	94.4 ± 17.2	106.4 ± 12.2	<0.0001	92.5 ± 16.3	100.8 ± 14.2	<0.0001
FVC (% predicted)	107.5 ± 14.6	111.0 ± 12.9	0.021	106.5 ± 15.3	111.6 ± 13.4	<0.0001	99.6 ± 15.9	105.4 ± 15.9	0.001
AHR (positive : negative : NA)	189 : 33 : 18	11 : 109 : 0	<0.0001	258 : 55 : 36	25 : 233 : 3	<0.0001	108 : 29 : 96	18 : 42 : 60	<0.0001
Blood IgE (UI/mL)	321.7 ± 935.8	133.3 ± 252.1	<0.0001	279.3 ± 808.9	87.0 ± 190.6	<0.0001	606.7 ± 1089.9	231.5 ± 375.8	<0.0001
Blood eosinophils (%)	3.6 ± 2.6	2.4 ± 1.6	<0.0001	3.4 ± 2.5	2.2 ± 1.6	<0.0001	3.8 ± 2.7	2.7 ± 1.7	<0.0001
Atopy (positive : negative : NA)	240 : 0 : 0	120 : 0 : 0	<0.0001	299 : 46 : 4	154 : 107 : 0	<0.0001	221 : 0 : 12	111 : 0 : 9	<0.0001
Smoking status (*n*) [%]									
Nonsmoker	166 [69.2]	85 [70.8]	0.001	225 [64.5]	190 [72.8]	0.002	155 [66.5]	54 [45.0]	0.001
Ex-smoker	69 [28.7]	23 [19.2]		110 [31.5]	52 [19.9]		37 [15.9]	33 [27.5]	
Smoker	5 [2.1]	12 [10.0]		14 [4.0]	19 [7.3]		37 [15.9]	29 [24.2]	

BMI, body mass index; FEV1, forced expiratory volume in 1 second; FVC, forced vital capacity; AHR, airway hyperresponsiveness, defined as provocative concentration of methacholine inducing a 20% fall in FEV1 (PC20) <8 mg/mL; and IgE, immunoglobulin E.

Continuous variables are expressed as mean ± SD. *P* values were calculated using *t*-test or chi-square tests as appropriate.

**Table 2:** The top 20 ranked SNPs in the pooled GWAS.

dbSNP ID	Chromosome	Silhouette score	Gene	Position
rs12418753	11p15.4	0.709	*NLRP14*	5′UTR
rs4487406	4q28.3	0.694		Intergenic
rs17655581	13q32.3	0.692		Intergenic
rs5904772	Xq27.3	0.689		Intergenic
rs11134480	5q34	0.686	*ODZ2*	Intronic
rs12881815	14q23.2	0.669	*SYNE2*	Exonic
rs12629883	3q28	0.666		Intergenic
rs10968574	9p21.1	0.661	*LINGO2*	Intronic
rs11600687	11q25	0.658		Intergenic
rs7980829	12q15	0.655		Intergenic
rs11956185	5q35.2	0.654		Intergenic
rs12070470	1p31.3	0.648	*IL23R*	Intronic
rs739337	8p22	0.647	*MTMR7*	Intronic
rs5970666	Xp22.11	0.646		Intergenic
rs881754	11q24.1	0.646		Intergenic
rs12493799	3q27.3	0.646		Intergenic
rs4808611	19p13.11	0.646	*NR2F6*	Intronic
rs7896493	10p14	0.644		Intergenic
rs2335562	13q12.12	0.643		Intergenic
rs17500510	6p21.32	0.636	*HLA_DQA2*	Intronic

**Table 3:** SNPs ranked in the top 2000 of the pooled GWAS that are near or within asthma candidate genes and selected for individual genotyping.

dbSNP ID	Chromosome	Rank^*∗*^	Silhouette score	Near candidate gene(s)
rs7576929	2q35	56	0.618	*SLC11A1*
rs3094738	6p21.33	85	0.593	*MICB*
rs6934016	6q25.1	100	0.585	*ESR1*
rs5250	14q12	115	0.577	*CMA1*
rs17016738	3p24.2	220	0.544	*RARB*
rs17453235	2q14.1	290	0.531	*DPP10*
rs7131715	12q13.12	305	0.528	*AQP5*
rs3097657	6p21.32	371	0.517	*HLA-DPB1*
rs2071596	6p21.33	373	0.517	*LTA, MICB, NCR3, TNF*
rs2858769	Xp11.23	515	0.502	*TIMP1*
rs9874200	3q26.2	562	0.497	*MECOM*
rs10932034	2q33.2	567	0.497	*ICOS*
rs2904774	6p21.33	601	0.494	*MICB*
rs17466945	1q32.1	626	0.492	*CHI3L1*
rs803010	14q22.1	723	0.484	*PTGDR, PTGER2*
rs1889371	14q32.2	742	0.482	*BDKRB2*
rs10864910	2q13	786	0.479	*IL1RN*
rs241423	6p21.32	811	0.478	*TAP1*
rs17160155	11q13.5	881	0.473	*LRRC32*
rs3135195	6p21.32	961	0.468	*HLA-DPB1*
rs3093665	6p21.33	970	0.467	*AIF1, LTA, NCR3, TNF*
rs17801353	12p13.31	981	0.466	*C3AR1*
rs6657275	1q41	1017	0.464	*TGFB2*

^*∗*^Ranking based on the pooled GWAS.

**Table 4:** Genetic association results for 38 SNPs tested by individual genotyping in 349 cases and 261 controls.

dbSNP ID	Chromosome	Minor allele	Freq in cases^*∗*^	Freq in controls^*∗*^	Major allele	*P* value^†^	Odd ratio
rs17655581	13q32.3	G	0.02	0.07	A	8*E* − 05	0.32
rs7980829	12q15	A	0.13	0.21	C	2*E* − 04	0.57
rs803010	14q22.1	A	0.30	0.21	G	4*E* − 04	1.60
rs10932034	2q33.2	A	0.25	0.17	G	0.001	1.59
rs10968574	9p21.1	G	0.10	0.05	A	0.002	2.07
rs17453235	2q14.1	G	0.13	0.08	A	0.005	1.71
rs12070470	1p31.3	G	0.12	0.07	A	0.005	1.78
rs881754	11q24.1	G	0.12	0.08	A	0.007	1.71
rs739337	8p22	G	0.05	0.02	A	0.008	2.54
rs11134480	5q34	G	0.13	0.09	A	0.012	1.61
rs17500510	6p21.32	A	0.14	0.09	G	0.018	1.55
rs2904774	6p21.33	G	0.03	0.01	A	0.019	3.43
rs7896493	10p14	A	0.13	0.09	G	0.019	1.55
rs6934016	6q25.1	A	0.14	0.10	C	0.020	1.53
rs12418753	11p15.4	A	0.04	0.02	G	0.021	2.30
rs3093665	6p21.33	C	0.03	0.01	A	0.021	2.62
rs17016738	3p24.2	A	0.30	0.24	G	0.023	1.35
rs17801353	12p13.31	C	0.40	0.34	A	0.028	1.30
rs9874200	3q26.2	G	0.11	0.07	A	0.029	1.58
rs5904772	Xq27.3	G	0.14	0.10	A	0.029	1.49
rs10864910	2q13	A	0.14	0.18	G	0.033	0.72
rs12629883	3q28	G	0.02	0.01	A	0.055	2.58
rs11956185	5q35.2	G	0.10	0.07	A	0.071	1.48
rs5250	14q12	A	0.13	0.09	G	0.082	1.39
rs6657275	1q41	G	0.32	0.27	A	0.088	1.24
rs3094738	6p21.33	G	0.08	0.06	A	0.099	1.47
rs3135195	6p21.32	C	0.06	0.04	A	0.129	1.53
rs7131715	12q13.12	G	0.09	0.07	A	0.130	1.39
rs4487406	4q28.3	G	0.08	0.06	A	0.190	1.36
rs3097657	6p21.32	G	0.05	0.04	A	0.190	1.45
rs2335562	13q12.12	A	0.18	0.16	G	0.218	1.21
rs5970666	Xp22.11	G	0.04	0.03	A	0.307	1.41
rs241423	6p21.32	A	0.04	0.06	G	0.311	0.76
rs4808611	19p13.11	A	0.17	0.15	G	0.312	1.17
rs17466945	1q32.1	C	0.11	0.09	A	0.316	1.22
rs2071596	6p21.33	A	0.15	0.14	G	0.453	1.13
rs17160155	11q13.5	G	0.14	0.12	A	0.488	1.13
rs11600687	11q25	A	0.09	0.09	G	0.774	1.06

^*∗*^Minor allele frequency in cases and controls.

^†^Sorted by *P* values.
