# Supplementary material for: Identification of Susceptibility Genes of Adult Asthma in French Canadian Women
Source: Can Respir J. 2016 May 4;2016:3564341. doi: 10.1155/2016/3564341 (PMC4904514; doi:10.1155/2016/3564341)
Supplement: Supplementary file 1 — Supplementary materials contain additional details about the methodologies. It also contains two figures showing the pool-GWAS design (Suppl. Figure 1) and the asthma genetic association results stratified by sex in the QCCCAC for the 38 genotyped SNPs (Suppl. Figure 2). Eight supplementary tables are also available showing results for 38 SNPs tested by individual genotyping in 299 allergic cases and 154 allergic controls (Suppl. Table 1), completed results for the 21 SNPs associated with asthma and SNPs in LD (Suppl. Table 2), most significant lung eQTL (Suppl. Table 3), in silico analyses results (Suppl. Tables 4 to 6), and results from the SLSJ asthma family collection (Suppl. Tables 7 and 8). [file 3564341.f1.zip › Supp Mat/Pooling_V16_Suppl_revision_Clean.docx]

**Supplementary Materials**

**IDENTIFICATION OF SUSCEPTIBILITY GENES OF ADULT ASTHMA IN FRENCH CANADIAN WOMEN**

Jean-Christophe Bérubé^1^, Nathalie Gaudreault^1^, Emilie Lavoie-Charland^1^, Laura Sbarra^1^, Cyndi Henry^1^, Anne-Marie Madore^2^, Peter D Paré^3,4^, Maarten van den Berge^5^, David Nickle^6^, Michel Laviolette^1^, Catherine Laprise^2^, Louis-Philippe Boulet^1^, Yohan Bossé^1,7^

# Methods

## DNA extraction and sample pooling

DNA was extracted using the QIAamp^®^ DNA Blood Midi Kit (QIAGEN) from buffy coat samples prepared the day of blood collection. DNA concentrations were assessed using Quant-iT™ Picogreen^®^ dsDNA Assay (Life Technologies). Stock DNA samples were stored at -80^o^C. Samples were diluted at 50 ng/µl in 96-well working plates and stored at -30^o^C. DNA from 240 women with allergic asthma was used to create the pool of cases. The second pool of DNA was prepared from 120 non-asthmatic and atopic women (controls). The same amounts of DNA (75 ng) from each sample were pipetted into their respective pool. DNA concentration of each pool was then confirmed by Quant-iT™ PicoGreen^®^ dsDNA Assay before genotyping.

**Pooled GWAS and analysis**

In this pooling-based GWAS, we used the silhouette ranking method to identify SNPs associated with asthma. Pearson et al. (Pearson et al. Am J Hum Genet 2007) demonstrated that the silhouette score was consistently the most effective method to rank SNPs in pooled-GWAS. Comparing different methods of ranking SNPs in pooling-based GWAS, Bossé et al. (Bossé et al. Hum Genet 2009) further validated that the silhouette score is the single best metric to use when less than 3,000 SNPs are considered for validation by individual genotyping.

## Individual genotyping and quality controls

Genotyping was performed using the two-color Illumina GoldenGate genotyping assay on VeraCode microbeads and read on the Illumina BeadXpress platform. We filtered out SNPs with call rates lower than 95%, monomorphic SNPs, and SNPs with a minor allele frequency lower than 1%. Clusters of SNPs with a p-value lower than P < 0.01 for Hardy-Weinberg equilibrium were visually inspected with the Illumina GenomeStudio software. After quality control, 38 out of the 43 SNPs remained. Five SNPs failed QC because of a low call rate (n=4) and deviating from Hardy-Weinberg equilibrium (n=1).

## In *silico* analyses

eQTLs were further evaluated using the Genotype-Tissue Expression project (GTEx) data ([1](#_ENREF_1)) integrated in HaploReg V4 ([2](#_ENREF_2)) and the collection of eQTLs integrated in RegulomeDB V1.1 ([3](#_ENREF_3)).

The LD patterns of the SNPs were obtained using the genotypes of 420 subjects recruited in the LAVAL cohort of the eQTL dataset. LD SNPs were identified using the PLINK 1.9 software ([4](#_ENREF_4)), by setting the R^2^ threshold at 0.8 and the limit distance to 500Kb. Together, it resulted in a set of 309 SNPs, henceforth referred as *LD* SNPs. The Phenotype-Genotype Integrator was also used to verify if any of these SNPs have been previously associated with asthma or other related traits by GWAS (http://www.ncbi.nlm.nih.gov/gap/phegeni).

Integrative tools were employed to annotate SNPs and identify putative functions. Tools used in this study are: Combined-Annotation-Dependent Depletion (CADD) V1.3 (http://cadd.gs.washington.edu/)([5](#_ENREF_5)), SNP Function Prediction (FuncPred) (http://snpinfo.niehs.nih.gov/snpinfo/snpfunc.htm)([6](#_ENREF_6)), RegulomeDB V1.1 (http://regulome.stanford.edu/)([3](#_ENREF_3)) and Haploreg V4 (http://compbio.mit.edu/HaploReg) ([2](#_ENREF_2)).

## Validation in the Saguenay-Lac-St-Jean (SLSJ) family collection for asthma

The SLSJ asthma family collection has been previously described ([7](#_ENREF_7)). This collection consists of French Canadian families ascertained through asthmatic probands. Genotyping was performed in 353 asthmatic allergic and non-asthmatic allergic women using the Illumina Human610-Quad BeadChip. To replicate genetic associations with 21 SNPs nominally associated with asthma in the QCCCAC, a quasi-likelihood score test was performed using the MQLS program (http://csg.sph.umich.edu/liang/MQLS/). This program allows a case-control analysis of binary traits with individuals who are related even in complex inbred pedigree ([8](#_ENREF_8)). SNPs were filtered out on the basis of the same criteria as the individual genotyping in the QCCCAC. SNPs missing from the genotyping array were tested through LD SNPs (R^2^ > 0.8).

# Supplementary Figures

## Supplementary Figure 1. GWAS on pooled DNA samples

## Supplementary Figure 2. Asthma genetic associations stratified by sex in the QCCCAC for the 38 genotyped SNPs.

# Supplementary Tables

**Supplementary Table 1** Genetic association results for 38 SNPs tested by individual genotyping in 299 allergic cases and 154 controls.

**Supplementary Table 2** Results for the 21 SNPs with p < 0.05 for association with asthma and those in LD, including information on the genomic context, the LD mapping, the pooled GWAS, the individual genotyping in all case and control women, the replication in SLSJ family collection and the *in silico* functional prediction. The 21 SNPs associated with asthma are in bold.

**Supplementary Table 3** The most significant eQTLs (P < 10E-5) from the lung eQTL dataset.

**Supplementary Table 4** FuncPred tool output.

**Supplementary Table 5** RegulomeDB V1.1 output.

**Supplementary Table 6** Haploreg V4 output.

**Supplementary Table 7** Replication in the SLSJ asthma family collection for SNPs directly genotyped.

**Supplementary Table 8** Replication in the SLSJ asthma family collection for proxy SNPs.

**References**

1. GTEx Consortium. The Genotype-Tissue Expression (GTEx) project. Nat Genet. 2013 Jun;45(6):580-5.

2. Ward LD, Kellis M. HaploReg: a resource for exploring chromatin states, conservation, and regulatory motif alterations within sets of genetically linked variants. Nucleic Acids Res. 2012 Jan;40(Database issue):D930-4.

3. Boyle AP, Hong EL, Hariharan M, et al. Annotation of functional variation in personal genomes using RegulomeDB. Genome Res. 2012 Sep;22(9):1790-7.

4. Chang CC, Chow CC, Tellier LC, Vattikuti S, Purcell SM, Lee JJ. Second-generation PLINK: rising to the challenge of larger and richer datasets. Gigascience. 2015;4:7.

5. Kircher M, Witten DM, Jain P, O'Roak BJ, Cooper GM, Shendure J. A general framework for estimating the relative pathogenicity of human genetic variants. Nat Genet. 2014 Mar;46(3):310-5.

6. Xu Z, Taylor JA. SNPinfo: integrating GWAS and candidate gene information into functional SNP selection for genetic association studies. Nucleic Acids Res. 2009 Jul;37(Web Server issue):W600-5.

7. Laprise C. The Saguenay-Lac-Saint-Jean asthma familial collection: the genetics of asthma in a young founder population. Genes Immun. 2014 Apr;15(4):247-55.

8. Thornton T, McPeek MS. Case-control association testing with related individuals: a more powerful quasi-likelihood score test. Am J Hum Genet. 2007 Aug;81(2):321-37.
