# Supplementary material for: Identification of Susceptibility Genes of Adult Asthma in French Canadian Women
Source: Can Respir J. 2016 May 4;2016:3564341. doi: 10.1155/2016/3564341 (PMC4904514; doi:10.1155/2016/3564341)
Supplement: Supplementary file 1 — Supplementary materials contain additional details about the methodologies. It also contains two figures showing the pool-GWAS design (Suppl. Figure 1) and the asthma genetic association results stratified by sex in the QCCCAC for the 38 genotyped SNPs (Suppl. Figure 2). Eight supplementary tables are also available showing results for 38 SNPs tested by individual genotyping in 299 allergic cases and 154 allergic controls (Suppl. Table 1), completed results for the 21 SNPs associated with asthma and SNPs in LD (Suppl. Table 2), most significant lung eQTL (Suppl. Table 3), in silico analyses results (Suppl. Tables 4 to 6), and results from the SLSJ asthma family collection (Suppl. Tables 7 and 8). [file 3564341.f1.zip › Supp Mat/SupplTable1_V2.docx]

## Supplementary Table 1 Genetic association results for 38 SNPs tested by individual genotyping in 299 allergic cases and 154 controls

| **dbSNP ID** | **Chromosome** | **Mean freq in cases pool*** | **Mean freq in controls pool*** | **Minor allele** | **Freq in cases*** | **Freq in controls*** | **Major allele** | **P-value^†^** | **Odd Ratio** |
| --- | --- | --- | --- | --- | --- | --- | --- | --- | --- |
| rs7896493 | 10p14 | 0.13 | 0.03 | A | 0.14 | 0.05 | G | 3.E-05 | 3.26 |
| rs7980829 | 12q15 | 0.10 | 0.26 | A | 0.13 | 0.23 | C | 3.E-05 | 0.47 |
| rs17655581 | 13q32.3 | 0.03 | 0.11 | G | 0.02 | 0.07 | A | 2.E-04 | 0.29 |
| rs12070470 | 1p31.3 | 0.19 | 0.05 | G | 0.12 | 0.05 | A | 5.E-04 | 2.70 |
| rs11134480 | 5q34 | 0.13 | 0.04 | G | 0.14 | 0.06 | A | 0.001 | 2.43 |
| rs10968574 | 9p21.1 | 0.18 | 0.06 | G | 0.11 | 0.04 | A | 0.001 | 2.69 |
| rs881754 | 11q24.1 | 0.17 | 0.08 | G | 0.13 | 0.06 | A | 0.001 | 2.36 |
| rs4487406 | 4q28.3 | 0.14 | 0.04 | G | 0.09 | 0.04 | A | 0.005 | 2.55 |
| rs803010 | 14q22.1 | 0.31 | 0.18 | A | 0.30 | 0.21 | G | 0.005 | 1.59 |
| rs4808611 | 19p13.11 | 0.28 | 0.15 | A | 0.17 | 0.10 | G | 0.006 | 1.80 |
| rs9874200 | 3q26.2 | 0.24 | 0.13 | G | 0.12 | 0.06 | A | 0.007 | 2.08 |
| rs6934016 | 6q25.1 | 0.08 | 0.01 | A | 0.15 | 0.09 | C | 0.007 | 1.85 |
| rs739337 | 8p22 | 0.13 | 0.06 | G | 0.05 | 0.01 | A | 0.007 | 3.84 |
| rs17801353 | 12p13.31 | 0.44 | 0.32 | C | 0.40 | 0.31 | A | 0.010 | 1.47 |
| rs2904774 | 6p21.33 | 0.20 | 0.11 | G | 0.03 | 0.00 | A | 0.011 | 8.89 |
| rs10932034 | 2q33.2 | 0.04 | 0.01 | A | 0.25 | 0.18 | G | 0.011 | 1.57 |
| rs17016738 | 3p24.2 | 0.08 | 0.01 | A | 0.31 | 0.23 | G | 0.012 | 1.50 |
| rs5250 | 14q12 | 0.36 | 0.23 | A | 0.14 | 0.08 | G | 0.012 | 1.83 |
| rs12418753 | 11p15.4 | 0.16 | 0.08 | A | 0.05 | 0.01 | G | 0.012 | 3.56 |
| rs11600687 | 11q25 | 0.11 | 0.03 | A | 0.10 | 0.05 | G | 0.013 | 2.08 |
| rs17466945 | 1q32.1 | 0.16 | 0.09 | C | 0.11 | 0.06 | A | 0.016 | 1.90 |
| rs11956185 | 5q35.2 | 0.17 | 0.06 | G | 0.10 | 0.05 | A | 0.017 | 1.98 |
| rs17160155 | 11q13.5 | 0.19 | 0.11 | G | 0.14 | 0.08 | A | 0.020 | 1.73 |
| rs17500510 | 6p21.32 | 0.17 | 0.07 | A | 0.14 | 0.08 | G | 0.021 | 1.72 |
| rs5904772 | Xq27.3 | 0.16 | 0.07 | G | 0.14 | 0.09 | A | 0.025 | 1.67 |
| rs3094738 | 6p21.33 | 0.14 | 0.05 | G | 0.09 | 0.05 | A | 0.026 | 1.93 |
| rs6657275 | 1q41 | 0.34 | 0.23 | G | 0.33 | 0.26 | A | 0.038 | 1.38 |
| rs12629883 | 3q28 | 0.04 | 0.00 | G | 0.03 | 0.01 | A | 0.040 | 4.16 |
| rs17453235 | 2q14.1 | 0.19 | 0.11 | G | 0.14 | 0.09 | A | 0.044 | 1.58 |
| rs5970666 | Xp22.11 | 0.06 | 0.00 | G | 0.04 | 0.02 | A | 0.055 | 2.52 |
| rs10864910 | 2q13 | 0.11 | 0.20 | A | 0.14 | 0.18 | G | 0.056 | 0.70 |
| rs2335562 | 13q12.12 | 0.23 | 0.11 | A | 0.19 | 0.14 | G | 0.071 | 1.42 |
| rs3093665 | 6p21.33 | 0.04 | 0.01 | C | 0.03 | 0.01 | A | 0.072 | 2.60 |
| rs241423 | 6p21.32 | 0.03 | 0.08 | A | 0.04 | 0.07 | G | 0.080 | 0.59 |
| rs7131715 | 12q13.12 | 0.13 | 0.07 | G | 0.09 | 0.06 | A | 0.086 | 1.62 |
| rs3135195 | 6p21.32 | 0.10 | 0.01 | C | 0.06 | 0.04 | A | 0.158 | 1.61 |
| rs3097657 | 6p21.32 | 0.06 | 0.00 | G | 0.06 | 0.04 | A | 0.187 | 1.56 |
| rs2071596 | 6p21.33 | 0.26 | 0.18 | A | 0.15 | 0.12 | G | 0.229 | 1.29 |

* Minor allele frequency in cases and controls.

^†^ Sorted by P values
